# African American women's perceptions of cancer clinical trials

**DOI:** 10.1002/cam4.284

**Published:** 2014-06-06

**Authors:** Lindsey Haynes-Maslow, Paul Godley, Lisa Dimartino, Brandolyn White, Janice Odom, Alan Richmond, William Carpenter

**Affiliations:** 1 Health Policy and Management, University of North Carolina at Chapel HillChapel Hill, North Carolina; 2 UNC-Lineberger Comprehensive Cancer CenterChapel Hill, North Carolina; 3 Crossworks, Inc.Rocky Mount, North Carolina; 4 North Carolina Institute of Minority Economic DevelopmentDurham, North Carolina; 5 Health Policy and Management, UNC Gillings School of Global Public HealthChapel Hill, North Carolina

**Keywords:** African Americans, cancer, clinical research, racial disparities, recruitment

## Abstract

Cancer clinical trials are important for resolving cancer health disparities for several reasons; however, clinical trial participation among African Americans is significantly lower than Caucasians. This study engaged focus groups of 82 female African American cancer survivors or cancer caregivers, including those in better resourced, more urban areas and less resourced, more rural areas. Informed by an integrated conceptual model, the focus groups examined perceptions of cancer clinical trials and identified leverage points that future interventions may use to improve enrollment rates. Study findings highlight variation in community knowledge regarding cancer clinical trials, and the importance of community education regarding clinical trials and overcoming historical stigma associated with clinical research specifically and the health care system more generally. Study participants commented on the centrality of churches in their communities, and thus the promise of the church as loci of such education. Findings also suggested the value of informed community leaders as community information sources, including community members who have a previous diagnosis of cancer and clinical trial experience. The sample size and location of the focus groups may limit the generalizability of the results. Since the women in the focus groups were either cancer survivors or caregivers, they may have different experiences than nonparticipants who lack the close connection with cancer. Trust in the health system and in one's physician was seen as important factors associated with patient willingness to enroll in clinical trials, and participants suggested that physicians who were compassionate and who engaged and educated their patients would build important trust requisite for patient participation in clinical trials.

## Introduction

Substantial cancer disparities exist between Caucasians and African Americans in the United States (US). African Americans are diagnosed with more advanced cancer, experience higher mortality rates, and have substantially lower 5-year survival rates than Caucasians 1. In North Carolina, minority women have a 49% greater breast cancer mortality rate than Caucasian women 2. Racial differences can largely be attributed to barriers for African Americans in accessing high-quality medical care and treatment 1,3–5.

Cancer clinical trials are important for resolving cancer health disparities for several reasons. In the short term, they are associated with high-quality, guideline-driven health care. In the longer term, heterogeneity of trial participants is important for the development of new interventions that are broadly effective, and not just effective in a subset of the population 6. Unfortunately, less than 5% of adult cancer patients are enrolled in a cancer clinical trial sponsored by the National Cancer Institute (NCI) 7. Among the 5% of adults participating in cancer clinical trials, less than 10% are African Americans 8. A recent study of North Carolina enrollment rates in NCI trials found that although the state's enrollment rates are comparable to national enrollment estimates, participation is lowest among African Americans. The racial disparity between Caucasians and minorities appears to be widening, and numerous North Carolina counties had no minority trial enrollment whatsoever 9.

One barrier to clinical trial enrollment is related to lack of access to the health care system 10, which often times disproportionately affects African Americans 11. Other factors that have been cited as barriers include time traveling to clinics and office visits, health literacy, and challenges navigating the health care system 12,13. Surveys examining clinical trial enrollment barriers among African Americans have shown mixed results. In a pilot survey expanding African American physician perceptions about clinical trials, 166 physician reported that low enrollment were due to lack of patient awareness and patient mistrust of the medical community 14. However, another study involving 70 African Americans revealed that the most important reason for not participating in clinical trials was health-related risks including side effects and interfering with current medications. This led the authors to conclude that African Americans were predominantly influenced by practical issues, rather than psychosocial perceptions 15.

While recent qualitative studies have focused on clinical trial enrollment and minorities, no studies have focused specifically on African American women and cancer clinical trial enrollment 16,17. The authors of this study recently conducted focus groups with African American prostate cancer survivors and their caregivers regarding their perceptions of cancer clinical research 16. Results showed that men were often confused about the relationship among clinical trials, treatment, and research, as well as apprehension to discuss health issues with a physician and overall mistrust in the medical system. The literature documents that men and women have different perspectives and approaches to health and health care, especially in the African American community. Additionally, cancer treatment and clinical trials differ substantially between women and men. For example, the most common cancer among African American women is breast cancer, accounting for 30% of all female cancer cases. Breast cancer treatment is very advanced, with innumerous combinations of therapies depending on the specific results of very advanced genetic and cellular tests. The most common cancer among African American men is prostate cancer, accounting for nearly 40% of all cancer cases, which has virtually no diagnostic tests that can inform treatment decisions, and treatment options are profoundly few.

Therefore, to better understand why African American women have low enrollment rates in clinical trials, focus groups were used to elicit African American women's thoughts, attitudes, and beliefs regarding cancer clinical trials. More specifically, our purpose was to determine the perceived barriers and facilitators to participating in cancer clinical trials. Using focus groups are advantageous because they provide information that would have not been obtained through surveys or interviews 29,30. Due to the varying health care norms and treatment options available for African American men and women, the authors felt it was necessary to focus only one gender, women.

## Methods

### Conceptual model

The study was guided by three conceptual models: the Lay Health Advisor model, Flaskerud and Winslow's vulnerable populations' framework, and the Behavioral Model for Vulnerable populations 18–20. This integrated model informed our understanding of the multiple, interrelated characteristics that influence African American women's willingness to participate in cancer clinical trials, including: (1) patient and caregiver-related characteristics (age, race, education, health preferences, uncertainty about research, transportation, childcare, time, and additional costs) 21–23; and (2) physician-related characteristics (scheduling appointments, protocol compliance issues, and access to and/or awareness of trials) 24–27.

### Setting

Between 2011 and 2012, eight focus groups were conducted across four counties in North Carolina (two focus groups per county): Guilford, Orange, Edgecombe, and Nash. These four counties were sampled from two regions, one in Central North Carolina and the other in Eastern North Carolina. These two regions were selected for this study to help assure diverse representation, as they are distinct from each other, and have different social and economic factors associated with them. Guilford and Orange counties, located in Central North Carolina, are comparatively better resourced, with two NCI Community Clinical Oncology Program Network (CCOP)-affiliated hospitals and one medical school. Nash and Edgecombe, located in Eastern North Carolina, are less resourced, with no CCOP hospitals or medical schools in a much more rural region of the state. From 2006 to 2010, minorities accounted for approximately 20% of the newly diagnosed cancer cases Guilford and Orange County, and 70% of newly diagnosed cancer cases in Nash and Edgecombe County 26.

### Participant recruitment and data collection

Inclusion criteria for participants were (1) 18 years or older (2) having a diagnosis of cancer (or a caregiver for a person with cancer), (3) not actively undergoing cancer treatment, and (4) having cognitive functioning sufficient to allow completion of the study. Focus group participants were recruited with the help of community contacts affiliated with the Carolina Community Network to Reduce Cancer Health Disparities (CCN II), a regional cancer network funded by the NCI Center to Reduce Cancer Health Disparities to reduce cancer disparities in North Carolina 31,32. Community contacts were individuals affiliated with nonprofit organizations, health care organizations, and faith-based organizations with previous cancer health disparities experience working with CCN II and the university. Community contacts used word-of-mouth and recruitment flyers to disseminate study information to potential focus group participants in their regional networks. Using community contacts to deliver study information via word-of-mouth and flyers is an effective recruitment strategy because they have established relationships with cancer survivors and caregivers and are able to quickly identify potential participants that may have an interest in the study.

Focus groups were held at community facilities (hospitals, churches, and community centers) recommended by community partners as being familiar to and conveniently located for participants. After determination of eligibility, participants completed a short survey regarding demographic and cancer-related questions. To test differences between cancer survivors and cancer caregivers, as well as differences between regions, (central vs. eastern North Carolina) *t*-tests for continuous variables and chi-squared tests for categorical variables were calculated. Due to the small sample size, *P* < 0.10 was considered statistically significant. Each focus group lasted approximately 90 min, and was conducted in collaboration with an independent research company. One African American female staff member from this company moderated all focus groups. Focus group discussions were digitally recorded and participants provided verbal consent to participate. Participants were compensated with a $60 gift card.

### Moderator guide and data analysis

The conceptual models directly informed the development of the focus group moderator guide. Focus group questions elicited discussions about perceptions of cancer clinical trial participation, practical barriers to participation, experience with clinical trials, medical decision making, and treatment preferences. To be more specific to the context of women's health, family roles, and female-specific cancers, the moderator guide and codebook were adapted from our previous qualitative study with African American men 33. Focus groups were transcribed verbatim and qualitatively analyzed using the software program Atlas.ti 6.0. Two members of the research team independently coded eight transcripts, adding new codes based on themes that emerged during the coding process, and reconciling codes upon completion of independent coding. Code summaries and memos for each focus group were written to reflect major themes and perceptions. The research team analyzed the final themes and perceptions within each focus group and between each focus groups. To determine significant differences between the focus groups, members of the research team calculated and compared code frequencies. Researchers also assessed baseline levels of participants' knowledge and understanding of cancer research terminology by tracking the phrases used by participants to describe terminology. Two community collaborators affiliated with CCN II also independently reviewed and provided thoughtful interpretation of findings and their perceptions regarding implications.

## Results

A total of 90 participants across four counties were recruited for eight focus groups. Eight participants did not show up for the focus groups; an 8.9% no-show rate. The final study sample included 82 African American women. Ages ranged from 21 to 85, with an average age of 57 (see Table 1).

**Table 1:** Characteristics of focus group participants (*N* = 82)

Characteristic	Number	Percent^1^	Group difference between survivors and caregivers (*P*-value)	Group difference between Eastern and Central regions (*P*-value)
Cancer experience			–	0.89
Survivor	37	0.451		
Caregiver	43	0.524		
Both	2	0.024		
Employment status			0.38	0.027**
Full time	21	0.26		
Part time	14	0.17		
Retired	28	0.34		
Do not work	19	0.23		
Education			0.71*	0.098*
Some high school	2	0.02		
High school	21	0.26		
Some college	38	0.46		
College	21	0.26		
Household income			0.73	0.014**
Less than $10,000	11	0.13		
$10,000–$19,999	13	0.16		
$20,000–$39,999	24	0.29		
$40,000 or more	22	0.27		
Did not answer	11	0.13		
Marital status			0.28	0.56
Married	28	0.34		
Separated	3	0.04		
Divorced	19	0.23		
Widowed	12	0.15		
Single	20	0.24		
Regular source of health care			0.093*	0.68
Yes	79	0.96		
No	3	0.04		

1 Percentages may not sum to 100 due to rounding.

* *P* < 0.10,

** *P* < 0.05.

Fifty-two percent of the participants were cancer caregivers, 45% were survivors, and 2% were both. Based on *t*-tests, survivors were more likely to be older than caregivers, 60.7 years versus 55.6 years, respectively (*P* < 0.05). Chi-squared tests showed that 64% of survivors were either retired or not working, compared to 51.1% of caregivers being retired or not working, however, this was not statistically significant (*P* = 0.38). Additionally, survivors had slightly lower educational achievements than caregivers, 41.0% had a high school degree or less compared to 15.6% (*P* < 0.10). The majority of the survivors had breast cancer (59%); ended treatment more than 6 months prior to the focus group (79%); did not participate in a cancer clinical trial (87%); and did not have their doctor talk to them about participating in a cancer clinical trial (67%) (see Table 2). In terms of geographic regions, participants living in Eastern regions were more likely to work or be retired (*P* < 0.05) and have higher incomes (*P* < 0.05) than participants living in Central regions. Additionally, participants living in Eastern regions were more likely to have some college education, but participants living in Central regions were more likely to be college graduates (*P* < 0.10).

**Table 2:** Cancer survivor characteristics (*N* = 39)

Characteristic	Number	Percent^1^
Cancer type		
Breast	23	0.59
Colon	5	0.13
Ovarian	3	0.08
Cervical	2	0.05
Lung	2	0.05
Skin	2	0.05
Hodgkin's	2	0.05
Non Hodgkin's	2	0.05
Leg cancer	1	0.03
Did not answer	1	0.03
Treatment ended		
More than 6 months	31	0.79
Less than 6 months	7	0.18
Did not answer	1	0.03
Have you ever participated in a cancer clinical trial?		
Yes	5	0.13
No	34	0.87
Did your doctor ever talk to you about participating in a cancer clinical trial?		
Yes	12	0.31
No	26	0.67
Not sure	1	0.03
If doctor talked with you about cancer clinical trial, did you participate?		
Yes	4	0.33
No	8	0.67

1 Percentages may not sum to 100 due to of rounding.

During data analysis, we found that themes followed a continuum of beliefs and perceptions that began with more conceptual, vague ideas, and spanned to more concrete and specific issues. The conceptual ideas were grounded in impersonal examples often based on historical/cultural contexts, while the concrete issues were more personal, practical examples based on contemporary contexts and experiences. We identified four dominant themes across all focus groups: knowledge and understanding of cancer research terminology, distrust in US medical system, the importance of physician trust, and importance of faith in decision-making process (see Table 3). These dominant themes provide insight into reasons for nonenrollment and may serve as leverage points for future efforts to improve African American women's participation in cancer clinical trials. In general, caregivers and survivors' responses to focus group questions did not differ widely.

**Table 3:** Summary of focus group themes

Theme	Illustrative quote	Total references across all focus groups^1^
Knowledge and understanding of cancer research terminology	“I think the research comes first and then the treatment because the research is gonna help you with the treatment.”	70
Importance of faith in decision-making process	“You have to have a holistic approach doctor…not only do they deal with the cancer; they deal with the spirituality. They believe in God, they believe in you being healed.”	35
Distrust in medical system	“I'm not from the South. You read so much on how they just played around with the black women, just gave them anything. Some of them sterile, some of them can't have children, so that trust issue is a big issue.”	25
Importance of physician trust	“I would [participate in a clinical trial] if my doctor recommended and I trust him, I would participate because I trust his medical advice.”	21

1 Total references were calculated based on the number of times participants made statements that were coded as the barriers.

### Knowledge and understanding of cancer research terminology

Literature suggests that cancer research terminology may be an issue when communicating with patients. Therefore, at the beginning of each focus group to gauge participants' understanding of and preferences for these terms, the moderator introduced various cancer research terms that are commonly used interchangeably. The moderator asked participants to describe in their own words definitions for “clinical research,” “medical research,” “research study,” “comparative research,” and “comparative examination,” and decide by consensus which they preferred to use during the rest of the focus group. Five of the eight focus groups preferred the term “medical research.” Participants' knowledge, understanding, and preferences for medical terms varied between the suburban/urban areas (Guilford and Orange county) compared to the rural areas (Nash and Edgecombe county).

In general, the Guilford and Orange county focus groups were knowledgeable about cancer research. Participants thought that comparing two different treatments was beneficial, such as the standard of care verses a treatment that is believed to be better. They felt that random assignment to the different treatment groups was fair and not unethical. As exemplified by one woman, “You've already been told up front that there's a chance. You've already been informed ahead of time that you may or may not receive the new one or the old one, so … You can't be upset about it.” Some participants felt that the “fairness” of receiving the old or new treatment should depend on an individual's case (e.g., cancer aggressiveness). Some felt that random assignment was seen as taking one's autonomy and ability to make their own decision about cancer treatment. Some women preferred receiving the old treatment versus the experimental or “new” treatment in a clinical trial because they thought that the old treatment was superior to the new. The majority of participants were not surprised that few cancer clinical trials use placebos. Everyone agreed that they did not like the idea of clinical trial that involved placebo and felt it was “deceptive.” Cancer treatment was discussed as being more certain than cancer research.

Compared to the Guilford and Orange focus groups, the Nash and Edgecombe focus groups were generally less knowledgeable regarding cancer research and terminology. Among group differences between the regions, the term “clinical trial” brought up more negative references such as “experimentation,” “guinea pig,” “trial and error”, and “last resort.” Some women felt that one benefit of participating in a clinical trial would be the access to a support group, which would not be available if they were receiving medical care that was not part of a trial. Using the term “medical research” elicited responses such as “finding a cure” and “thorough exam.” As one woman commented, “the more research, the more possibilities there are to finding a cure….” In these groups, there was a general lack of knowledge about the concept of random assignment and the need for comparing two different treatments. After the moderator explained random assignment to participants, they expressed concern about not knowing which treatment they would receive, but thought the idea of random assignment was fair.

### Distrust in medical system

There remains widespread distrust and hesitation among African Americans with respect to medical research and trials. As one woman commented, “With medical research and being African Americans I know that we were used a long time ago for that, and that's why I think we're so afraid of it now because they used us in the past and we don't trust anyone.” Across all focus groups, when asked about clinical trials, medical research, and research studies, multiple women expressed their concerns about experimentation and referenced the Tuskegee Experiment. When asked what information participants would need to know before deciding whether to participate in cancer medical research, the following conversation arose:

Participant: *As a black woman, I need to know I'm not being used as a guinea pig or the financial aspects sometimes comes into play with my race in our all-black communities*.

Moderator: *How would you know that you're not being used as a guinea pig?*

Participant: *Bringing us together as races, not separating us or on applications to sign up that my ethnic background is not asked for*.

Moderator: *So if your ethnic background is asked for, what I think I hear you saying is that you have this inkling in your mind that they're using you as a guinea pig?*

Participant: *Yes*.

When asked if they would feel more comfortable participating in a clinical trial knowing that all cancer treatments underwent clinical trials, one woman said, “It is [important] because we as a race are automatically standoffish and so afraid from past things. We all know about the Tuskegee … and we just don't feel comfortable participating in things, and it's for lack of knowledge.”

The importance of good provider communication and being informed was highlighted in all focus groups as relevant to both the health care and clinical research context. For example, one woman whose husband participated in a clinical trial had their oncologist discuss the entire process with them and offer in-depth consultation. Because of the oncologist's willingness to take time and discuss the trial, the woman said, “…that's why I say research is good and everything is so different now. Now, they have chemo class. You actually go to class now before you start your treatment and when you get there, there's a nurse and she knows all about you, she has all your papers there…And they go over everything, and that just makes it so much easier. They give you so much more information now.” In sum, across all focus groups, women talked about the importance of trusting their physicians and how this could affect their willingness to participate in clinical trials. Overall, women who participated in a clinical trial or had family members participate were more likely to be more trusting of the medical system than women who had no experience with cancer clinical trials.

### Importance of physician trust

Trusting a physician was associated with having an established, existing relationship, and having a physician that was caring and compassionate. When asked how participants would feel if their physician were to discuss a cancer research study as a form of cancer treatment, one woman captured the perspectives of several participants when she responded, “It shows that he has some compassion. He's giving you all these options and you make a decision from the options that you have been given. Some doctors are so cut-and-dry… if he sits down and he goes through all of this, I would feel more comfortable and I would more likely stick with him and go to whoever he refer me to.”

Physician compensation was generally a problem for the majority of focus groups and was often tied to trusting the physician. Most participants felt that if the physician was receiving compensation for offering clinical trials, then it would decrease their willingness to participate. When asked how physician compensation would affect their willingness to participate, one woman responded, “If he's being compensated then he might not even care about me.” This being said, the Guilford and Orange county focus groups tended to not see compensation as unethical as long as the physician was forthcoming about it. “They get paid anyway … If they're going to come up with a cure, I'm for it.” One woman saw compensation as positively affecting her willingness to participate, “I think that the doctor, if he's going to get that check, he's going be diligent in making sure that he does everything that he needs to do.”

### Importance of faith in the decision-making process

One theme that transcended focus group locations was the importance of faith in the cancer care decision-making process. Across all groups, multiple references about the intersection between physicians and faith were brought up. As one cancer survivor commented about her diagnosis, “I just left it in my doctor's hands and in God's hands.” Another woman told a story about her physician praying with her and how she believed it helped her health outcome. One caregiver talked about how she would pray to God to “instill everything in that doctor” to provide good care. Other women discussed the importance of having a spiritual physician.

The importance of faith also came up in regards to cancer treatment and cancer clinical trials. As characterized by one survivor whose cancer had just returned, “What do I have to lose, after I pray and He makes that decision? I'm at the point in my life now where I would probably say ‘bring it on.’” In contrast to the cancer survivor's willingness to participate in a clinical trial, a survivor responded, “I have a higher power, so I consider myself healed. With me, I would never participate in a clinical trial.”

Further reflecting on the centrality of faith within the context of clinical trial participation among African American women, all of the focus group participants agreed that posting information at churches would be a good avenue for informing people about participating in clinical studies, other than the doctor's office. Women suggested posting flyers on church bulletins, placing inserts in programs, sending emails through church listservs, and having speakers talk to the congregation. One woman talked about the importance of having study information at church: “They should reach out and come. I mean, 80 percent of us as a group, as a culture, you could reach us at church.”

## Discussion

The purpose of this study was to examine African American women's thoughts, attitudes, and beliefs regarding cancer clinical trials, including perceived barriers and facilitators to participation. In this study, many themes and issues arose that are consistent with other studies 14,15,31–33. However, the important issue about this study's findings is that lack of knowledge, distrust in medical system, trust in physicians, and the importance of faith are *still very prominent themes* in African American women's lives today. This leads the authors to conclude that much is still required to be done when addressing the health disparities between African Americans and Whites. Several of the focus groups offered recommendations and strategies to address this gap.

While several studies highlight the importance of addressing tangible barriers 12,14,15, such as cost, health insurance, transportation, and child care, in clinical trial participation, participants in this study felt these barriers were not prominent themes. Some participants mentioned cost as a barrier to participation, in terms of health insurance and transportation, but many women said they would do “whatever it takes” to get the best care to survive. One woman said that cost and transportation would not be a barrier to participation because “You just want to get better. You want to live.”

Across the eight focus groups, the level of knowledge and understanding with respect to cancer medical research varied. The more rural focus groups were generally less knowledgeable than the urban focus groups regarding cancer research and terminology. Additionally, educational opportunities for participants in their physician's office also differed depending on the physician, clinic, or hospital. While some patients had the opportunity to discuss clinical trial information one-on-one with a nurse educator, others were handed a brochure and asked to call if they had questions. The fact that participants chose different terminology in focus groups to discuss cancer clinical trials is evidence that information being described to them lacks consistency. One strategy that could potentially be used to address this inconsistency is through patient navigators. Patient navigators are individuals who advocate on behalf of the patient, and assist them with making informed choices by addressing any confusion or misperceptions about clinical trial logistics 17. In a recent study examining the effectiveness of using patient navigators among Chinese patients with breast and gynecologic cancer, researchers found that navigators improved patient's knowledge of cancer clinical trials and trial participation 36. Our study suggests that more education and one-on-one time with potential cancer clinical trial participants could increase enrollment. Due to differences across geographic settings, researchers could consider offering more education and resources to women in rural areas than urban areas.

In terms of overcoming stigmas and historically founded suspicions of the US medical system with respect to research and clinical trials, many participants cited the importance of education, physician compassion, and communication 37,38. Physicians are the first point of contact for referring patients to cancer clinical trials, and it is important for patients that their physicians sanction that referral. A majority of the focus group participants expressed a desire for their physicians to be compassionate and considerate, to be transparent with regard to their remuneration, and to help them understand the information being discussed. This finding is consistent with a similar study conducted by the authors with African American men in North Carolina in which physician trust was crucial in considering cancer clinical trial participation 16. One theme among the men that was not present in women was the role of gender and social norms discussing health information and visiting the doctor. This not only highlights the importance physician trust, but the importance of cultural and social sensitivity when treating gender-specific minority patients 39. Based on the male/female differences in norms regarding health and health care as discussed in the introduction, this study's results supports the authors' decision to differentiate the focus groups by gender.

One strategy suggested by participants for educating African Americans about clinical trials is through the church. Focus group participants all indicated the centrality of the church in their community and advocated for its use in health education. Church-based interventions have long been recognized as effective methods for improving general health behaviors among African Americans 17,40–42. While the concept of church and faith did not arise in the African American men's focus groups, based on this study's finding, we believe that the church can be effective in communicating information about cancer clinical trials to women. This study also suggests that other opportunities to educate African American women about cancer clinical trials are through engaging with community leaders, support groups, and cancer survivors. Women who have participated in clinical trials may also be an appropriate avenue for communicating information to potential participants, as they can offer a personal perspective that may reach other women more effectively. A recent study tested the effectiveness of a 15-min, culturally targeted video involving unscripted narratives of African American patients discussing their attitudes and experiences with clinical trials following a cancer diagnosis. African American patients who watched the video had a 34% increase in the likelihood of enrolling in a clinical trial. The idea of using past participants for cancer clinical trial recruitment is not widely used, but shows promise 43.

This study has several limitations. First, the sample size and location of the focus groups may limit the generalizability of the results. Second, since the women in the focus groups were either cancer survivors or caregivers, they may have different experiences than nonparticipants who lack the close connection with cancer. Finally, characteristics of people who agreed to participate in this study may be different from those who did not agree to participate, including that nonparticipants may be less knowledgeable about cancer clinical trials. Future studies with African Americans should be conducted to clarify any differences among these groups, and also to validate the perceptions of cancer clinical trials presented in this study.

Our findings offer several strategies for increasing clinical trial enrollment among African American women in North Carolina, including continued community education and interventions within the church setting. To combat long-held historically based concerns about medical research, the US health care system needs to ensure that minority patients' interactions with the health care system are positive, and promote the establishment of a trusting relationship with physicians through open, honest information about cancer clinical trials.
